# Paclitaxel Drug Delivery Systems: Focus on Nanocrystals’ Surface Modifications

**DOI:** 10.3390/polym14040658

**Published:** 2022-02-09

**Authors:** Razan Haddad, Nasr Alrabadi, Bashar Altaani, Tonglei Li

**Affiliations:** 1 Department of Pharmaceutical Technology, Faculty of Pharmacy, Jordan University of Science and Technology, Irbid 22110, Jordan; altaani@just.edu.jo; 2 Department of Pharmacology, Faculty of Medicine, Jordan University of Science and Technology, Irbid 22110, Jordan; 3 Department of Industrial and Physical Pharmacy, Purdue University, West Lafayette, IN 47907, USA; tonglei@purdue.edu

**Keywords:** paclitaxel, nanocrystals, surface modification, chemotherapy, cancer, drug delivery, nanotechnology

## Abstract

Paclitaxel (PTX) is a chemotherapeutic agent that belongs to the taxane family and which was approved to treat various kinds of cancers including breast cancer, ovarian cancer, advanced non-small-cell lung cancer, and acquired immunodeficiency syndrome (AIDS)-related Kaposi’s sarcoma. Several delivery systems for PTX have been developed to enhance its solubility and pharmacological properties involving liposomes, nanoparticles, microparticles, micelles, cosolvent methods, and the complexation with cyclodextrins and other materials that are summarized in this article. Specifically, this review discusses deeply the developed paclitaxel nanocrystal formulations. As PTX is a hydrophobic drug with inferior water solubility properties, which are improved a lot by nanocrystal formulation. Based on that, many studies employed nano-crystallization techniques not only to improve the oral delivery of PTX, but IV, intraperitoneal (IP), and local and intertumoral delivery systems were also developed. Additionally, superior and interesting properties of PTX NCs were achieved by performing additional modifications to the NCs, such as stabilization with surfactants and coating with polymers. This review summarizes these delivery systems by shedding light on their route of administration, the methods used in the preparation and modifications, the in vitro or in vivo models used, and the advantages obtained based on the developed formulations.

## 1. Introduction

Currently, cancer is considered a serious disease that is globally widespread, and it is one of the most life-threatening illnesses [1], accounting for about 10 million deaths in 2020 [2]. Additionally, the economic burden of this disease is enormous, and it is anticipated to increase in the future [3,4]. On the other hand, chemotherapeutic agents are considered effective at fighting cancer and preventing its development and progress [5]. However, there is still an urgent need for more therapeutic options or strategies to improve the currently available treatments in terms of safety and efficacy.

The improvements of chemotherapeutic agents mainly depend on two research lines [5]. The first one is related to explaining cancer-specific mechanisms and molecular targets, such as signal transduction inhibitors concerning essential processes of cells such as growth, survival, and differentiation. These substances may have the ability to prevent the injuries caused by cancer cells, including proliferation and tissue invasion [6]. The second line is considering the enhancement of the available cytotoxic drugs which act on abundant targets (e.g., DNA or tubulin) [5]. These cytotoxic drugs are either natural products or their derivatives obtained from plants, marine species, and microorganisms, but unfortunately, these drugs are still toxic to normal cells [7]. Therefore, the improvement of their efficacy and safety is always warranted.

Eventually, many anticancer agents were obtained. but most of them are inefficient and cause severe side effects. Therefore, there is an emerging need to develop new therapeutic agents or delivery approaches. Several drug delivery systems based on nanotechnology modalities have been obtained for different anticancer drugs such as solid lipid nanoparticles, liposomes, micelles, polymeric nanoparticles, nano-emulsions, implants, and nanocrystals [8,9,10]. All these approaches are aimed at either enhancing the efficacy or reducing the side effects of the currently available chemotherapeutic agents. Finding novel and appropriate drug delivery systems is crucial, especially for chemotherapies where intravenous delivery remains the main route used for drug administration [8]. This returns to the fact that most anticancer drugs have low solubility or gastrointestinal tract (GIT) toxic side effects over oral administration, which in turn can reduce their oral absorption below the therapeutic effective levels [10].

## 2. Paclitaxel

Paclitaxel (PTX) is an important chemotherapeutic agent that belongs to the taxane family. Taxanes were initially obtained from plants of the genus *Taxus*. PTX was first derived from the bark of the Pacific yew (*Taxus brevifolia*), which is an evergreen tree and small to medium in size and also known as western yew, native to the Pacific Northwest of North America [11,12]. PTX was approved by the United States (US) Food and Drug Administration (FDA) to treat various kinds of cancers including breast cancer, ovarian cancer, advanced non-small-cell lung cancer, and acquired immunodeficiency syndrome (AIDS)-related Kaposi’s sarcoma [13]. In general, PTX is not well tolerated and related to serious adverse drug effects such as hypersensitivity reactions, hematological toxicity, peripheral sensory neuropathy, and myalgia or arthralgia [13], even though PTX has been used for two decades either as a single drug or in combination with other chemotherapeutics.

The antitumor activity of paclitaxel comes from its high binding affinity to microtubules, stabilizing and improving the polymerization of tubulin and destruction of the dynamics of the spindle microtubule [14,15]. Such activities provide effective inhibition of cell mitosis, intracellular transport, and motility, which end up with cell death by apoptosis. However, the clinical developments of the natural form of paclitaxel have been restricted due to its physicochemical properties, particularly its very low solubility [16]. Additionally, the absence of modifiable functional moieties in its structure makes the chemical alteration of the natural paclitaxel very complicated when attempting to enhance its solubility [17]. Considering that, the selection of a proper delivery system to paclitaxel is considered very crucial to improving its clinical development, safety, and efficiency.

**Figure 1:**
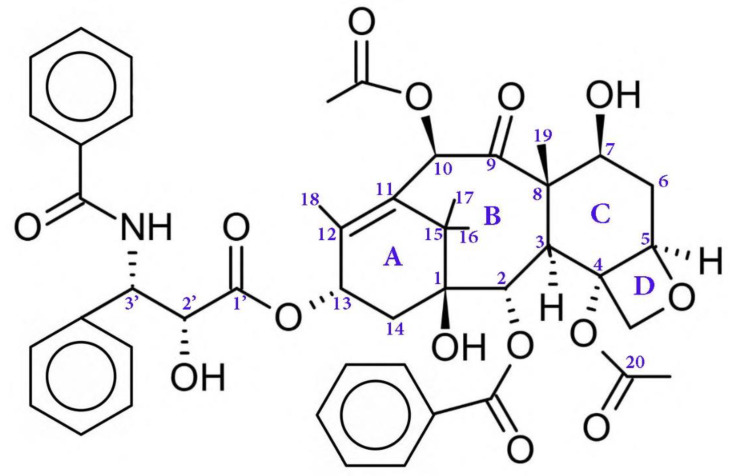
The chemical structure of a PTX drug.

Regarding the chemical structure of PTX (Figure 1), its 20-carbon compound (C_20_) belongs to the diterpene class of natural compounds [18]. The anticancer activity is mainly recognized for ring A, ring D (the oxetane ring), the C2 benzoyl group, and some components such as the C3′ amide-acyl group and the OH group at C2′, which attaches on the side chain to C13 [19]. On the other hand, other groups slightly affect the therapeutic activity of PTX such as the carbonyl group on C9 and the acetyl group on C10. Moreover, the specified conformation of the paclitaxel molecule is provided by the acetyl group [19].

PTX has poor aqueous solubility, low permeability, and as it is a P-gp substrate, it also has limited capabilities for being delivered via the oral route [20,21,22,23]. The poor permeability of PTX is related to the following molecular factors: its molecular weight is more than 500, the hydrogen bond acceptor (HBA) is greater than 10, and the polar surface area (PSA) is more than 140 A^2^, which results in a permeability coefficient value in the range of 10^−6^ cm/s [24,25]. Therefore, PTX is administered parentally via the intravenous (IV) route with a suitable cosolvent (cremophor EL and ethanol), which unfortunately ends up with several direct, problematic, adverse effects such as acro-anesthesia and neurovirulence, causing pain and high cost [26,27,28,29].

## 3. PTX Formulations

To improve the benefit and delivery of PTX, several formulations have been developed. The most commonly used delivery system is a cosolvent strategy based on a 50:50 mixture of ethanol and Cremophor EL™ (a polyoxyethylated castor oil). Taxol^®^ is the first generic product of paclitaxel, and it consists of this cosolvent mixture. Although this method overcomes the problem of solubility, Cremophor EL has been associated with non-linear pharmacokinetics and serious and dose-limiting toxicities, such as hypersensitivity, neurotoxicity, and nephrotoxicity [11]. Due to these adverse effects, Taxol^®^ is given slowly in 135- or 175-mg/m^2^ doses by infusion over 3–24 h every 3 weeks [30,31].

Abraxane™ is another marketed drug of PTX, which was produced by Abraxis BioScience (later obtained by the Celgene company) and approved by the FDA in 2005 [32]. The formulation of PTX in this product is performed with human serum albumin (HSA) [33]. HSA is the most abundant plasma protein in the blood, with a large half-life that reaches up to 19 h and which can bind hydrophobic substances irreversibly, transport them through the body, and deliver them to the cell surface [34]. Additionally, HSA plays a significant role in cellular uptake and transcytosis, as it is bound to gp60 and other proteins which are highly expressed in malignant cells, such as secreted proteins acidic and rich in cysteine (SPARC). Nevertheless, it is still ambiguous how exactly HAS improved the biological response of PTX. However, it is significantly clear that the removal of Cremphor EL contributes to the ability to administer a higher dose of PTX with an analogous toxicity [35]. Moreover, Abraxane™ has a linear pharmacokinetic profile and a higher intratumoral concentration by 33% in comparison with Taxol^®^, based on results obtained by the Abraxis BioSciences company [36].

Another marketed drug of PTX is Lipusu™, which was formulated by Luye Pharmaceutical Co. Ltd. and approved in China in 2003. It is a liposome composed of PTX, lecithin, and cholesterol. In comparison with Taxol^®^, Lipusu™ has similar activities toward breast cancer, non-small cell lung, and gastric cancer but with considerably lesser side effects [37,38].

Finally, Genexol-PM™ is marketed by Samyang Corporation and was approved in South Korea in 2007. It is composed of PTX and poly (ethylene glycol)-b-poly (lactic acid) (PEG-b-PLA) block copolymers. Clinical studies showed that Genexol-PM™ has dose-dependent pharmacokinetics and good tolerance, especially for patients with advanced pancreatic cancer or metastatic breast cancer [39,40,41].

## 4. Drug Delivery of PTX

**Figure 2:**
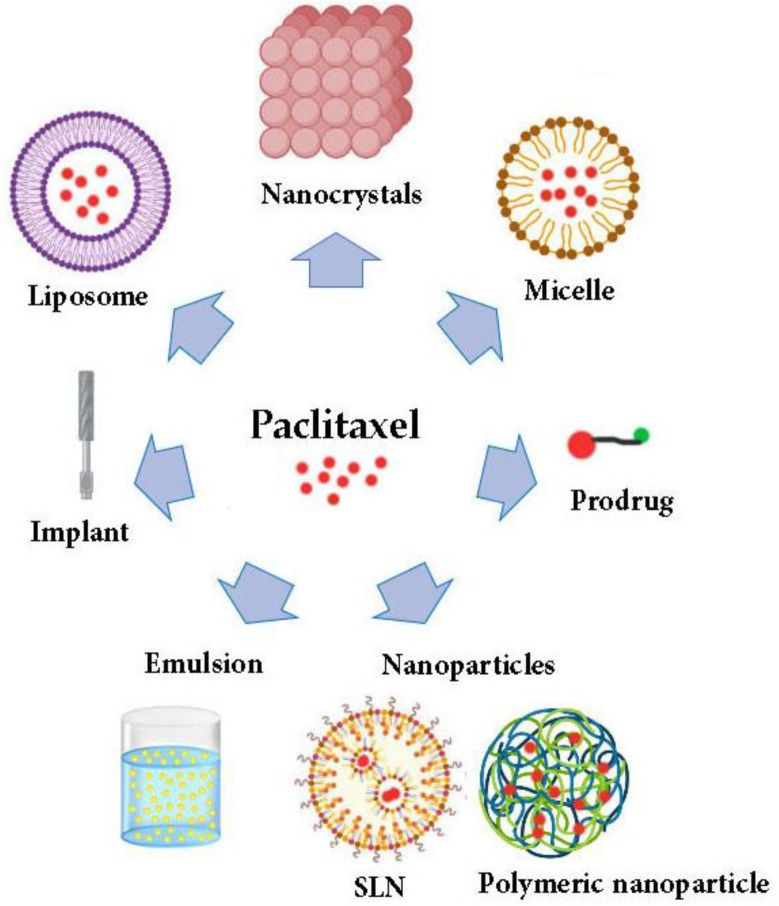
The most common developed strategies to improve the delivery of paclitaxel drugs. SLN: solid lipid nanoparticles.

As previously mentioned, the physicochemical properties and the nature of PTX complicated its formulations. Consequently, several delivery systems for PTX have been developed to enhance its solubility and pharmacological properties involving micelles, liposomes, nanoparticles, the prodrug approach, emulsions, implants, and nanocrystals [42,43,44,45]. Figure 2 summarizes the most common strategies utilized for PTX delivery systems.

### 4.1. Micelles

Generally, micelles consist of polar heads that encounter the outside aqueous environment and non-polar tails, which form the interior hydrophobic core. Above the critical micelle concentration, micelles spontaneously form, and drugs with low solubility encapsulate efficiently in the lipidic core [46,47]. The properties of micelles and the hydrophobic regions can be modified and tailored by using various polymer structures [48]. The targeted delivery of PTX micelles developed using an Asn-Gly-Arg (NGR) peptide, which covalently bonds to PEG chains to deliver PTX through a brain tumor [49]. Moreover, the oral delivery of PTX was obtained using multi-functional chitosan polymeric micelles [50]. The development of redox-sensitive PEG2000-S-S-PTX micelles resulted in a reduction of PTX cytotoxicity in ovarian and breast cancer cells [51].

### 4.2. Liposomes

The liposome is a spherical structure with a membrane composed of a single or multiple phospholipid bilayers. It has an aqueous core to encapsulate hydrophilic drugs, while the hydrophobic drug can be loaded in the region of the bilayer membrane. Liposomes have been used to deliver PTX because they showed that they can enhance solubility and efficacy by modulating its pharmacokinetic properties. Additionally, the used excipients are clinically approved [52]. Lipusu^TM^ is the first injected PTX liposome, and it has been used in China to treat non-small cell lung cancer, breast cancer, and other cancers [19]. It maintains the original activity of PTX but with a significant reduction in the side effects. Moreover, LEP-ETU is another liposome loaded with PTX. Based on the phase I study, LEP-ETU showed little difference in the pharmacokinetic properties in comparison to Taxol^®^ while being safer at higher doses [53].

Long-term instability is the main obstacle related to liposomes. Despite liposomes having the ability to deliver cytotoxic compounds to certain tissues, they can be eliminated by the mononuclear phagocytic system (MPS) in the spleen and liver [54]. Interestingly, the average circulation time of liposomes can be enhanced by 10 folders with PEGylation, which results in an improvement of the half-life of PTX and its antitumor properties [55,56,57]. The PEGylated liposome can be also modified by active targeting strategies to improve its efficacy [58,59]. This can be obtained by covalently binding species to the surface of the liposomes, such as the peptides [60], proteins [61], and tissue-specific antibodies [62]. Specifically, a multifunctional peptide was incorporated to the surface of the liposomes loaded with PTX, which improved its targeting activity and also its efficacy [59]. Moreover, triphenylphosphonium (TPP) was incorporated into the surface of the PEGylated PTX liposomes, which consequently enhanced their cytotoxicity and antitumor efficacy and provided efficient mitochondrial targeting in cancer cells [58]. Moreover, PTX loaded to a pH-sensitive lipid that was incorporated into a liposomal membrane prevented liposome degradation by lysozymes and consequently caused more suppression of tumors by providing more PTX accumulation at pH of 7.4 instead of 5.5 [63,64].

### 4.3. Nanoparticles

#### 4.3.1. Solid Lipid Nanoparticles

Generally, solid lipid nanoparticles (SLN) are obtained from solid lipids, such as complex glyceride, highly purified triglycerides, and waxes [65]. Several kinds of lipids and surfactants can be used for SLN production and engineering. More specifically, lipids such as phospholipids and glycerides and surfactants such as tween 80, sodium glycolate, lecithin, and poloxamer 188 are considered suitable for IV injection [66]. There are several advantages related to the SLN, such as the simplicity of the preparation method and the scaling up, biocompatibility, stability, low cost, low toxicity, controlled drug release, and versatile chemistry [52].

To obtain high drug loading and the slow release of PTX, SLN should obtain high drug solubility and miscibility [67]. The cellular uptake and cytotoxicity properties of PTX-loaded SLN can be vary based on the lipid materials used. For instance, studies showed that the cellular uptake of SLN was concentration- and time-dependent and related to the melting point of the lipidic materials, the length of its hydrocarbon chain, and the particle size [68,69,70]. PTX-loaded PEGylated steric acid SLN proved to have a high cellular uptake and up to 10-fold greater cytotoxicity in comparison with PTX. Moreover, SLN showed an ability to affect P-gp-mediated multidrug resistance (MDR), as PTX loaded SLN provided an inhibition of P-gp activity and a rapid depletion of ATP [71,72].

As with the other noncompaction systems, surface modification of the particles by different chemical moieties is useful for obtaining prolonged SLN circulation by avoiding the clearance with the reticuloendothelial system (RES) [52].

#### 4.3.2. Polymeric Nanoparticles

Polymers have been used in nanoparticle preparation to provide them with suitable properties and characteristics. Examples of some of the polymers that are commonly used in developing paclitaxel nanoparticles are poly (lactic-co-glycolic acid) (PLGA) and chitosan, which will be discussed in the following sections.

##### Poly Lactic-co-Glycolic Acid (PLGA)

Poly (lactic-co-glycolic acid) (PLGA) is a biocompatible, biodegradable, nontoxic synthetic polymer derived from poly (lactic acid) (PLA) and poly (glycolic acid) (PGA) [73,74]. It has been approved by the US Food and Drug Administration (FDA) for drug delivery, as it has superior properties in the delivery of many therapeutic agents. PLGA is a very useful and successful polymer in nanomedicine and the nano-delivery of drugs. In addition, it has a favorable ability to target tumors and DNA [73,75,76,77]. PLGA is available commercially with various molecular weights and copolymer ratios. Based on that, the duration of the degradation can vary, as can the release time. Glycolic acid is more hydrophilic than lactic acid, and thus PLGA with higher glycolic acid is more hydrophilic and can adsorb water more and degrade faster [78,79]. The loading of PTX to PLGA nanoparticles has been obtained by various methods such as emulsion solvent evaporation [77], interfacial deposition methods [80], and the nanoprecipitation method [81]. Studies showed that PTX loaded to the PLGA nanoparticles had superior antitumor properties and efficacy in comparison with Taxol^®^ [81,82]. Moreover, surface modification of the nanoparticles has a crucial impact on their properties, such as efficacy and targeting. The delivery of PTX was improved by surface modification of PLGA nanoparticles with albumin, as the circulation time of these nanoparticles in the blood was increased even as it became more toxic in the in vitro study [83]. The targeted delivery of PTX to breast cancer cells was developed by loading it into PLGA nanoparticles coated with hyaluronic acid (HA), and the results showed that the cellular uptake was increased using this system [84]. Moreover, PTX has been loaded to lipid PLGA hybrid nanoparticles, and the results showed that the release profile was affected with this lipid coat. Also, these nanoparticles provided a prolongation in the circulation time in the blood [85].

##### Chitosan

Chitosan is a natural polysaccharide polymer produced by the diacylation of chitin. It has many attractive properties such as non-toxicity, biocompatibility, biodegradability, and bio-adhesivity, which necessitates its use in drug delivery [86,87]. The solubility of chitosan in acidic solutions and its limited solubility in biological solutions (pH 7.4) are considered the main drawbacks of its application in drug delivery. Lately, many chitosan derivatives have been prepared by adding various hydrophobic or hydrophilic groups to the chitosan structure [88,89]. Moreover, studies showed that chitosan has antitumor properties, and it can affect the cancer cells by interfering with its metabolism, inhibiting its growth, or inducing its apoptosis [90].

Chitosan has been introduced to many PTX delivery systems, and it improved various aspects (e.g., decreasing the toxicity and enhancing the efficiency and targeting capabilities) [91,92,93]. A study showed that the PTX-loaded micelle based on N-octyl-O-sulfate chitosan (OSC), which is a novel derivative of water-soluble chitosan used for the delivery of PTX, has superior toxic properties, as lower side effects were observed, and the AUC was about 3.5 lower than the marketed drug Taxol^®^ while preserving the antitumor efficacy at equivalent doses [94]. Additionally, other studies showed that the targeted delivery of PTX chitosan nanoparticles had been achieved in combination with other polymers such as PEGylated chitosan nanoparticles grafted with Arg-Gly-Asp (RGD) [92], poly NIPAAm [95], transferrin [96], and biotinylated N-palmitoyl chitosan [97].

### 4.4. Prodrug Approach

Prodrugs are derivatives of a drug molecule that can be transformed chemically or enzymatically in the body to release the active ingredient that possesses pharmacological effects [98]. Differing from other delivery systems or formulations, prodrugs are usually formulated by chemical linkage with proper quality control and less variation from batch to batch. Generally, prodrugs are developed to overcome problems related to the parent drug itself, such as poor aqueous solubility, limited permeability, inadequate oral absorption and delivery, non-targeting, and toxic side effects [99,100].

The PTX prodrug is usually fabricated at the carbon no. 123; 2′or 7-OH group [100]. PTX prodrugs are constructed using various strategies, such as polymer-based prodrugs, which are formulated using polymers such as PEG [101], PLA [102,103,104], poly(amidoamine) [PAMAM] [105], N-(2-hydroxypropyl) methacry’lamide (HPMA) [106], and poly(L-glutamic acid) (PGA) [107]. Moreover, a protein-based prodrug of PTX has been developed using different proteins, such as the marketed product Abraxane™, which is tumor-targeted and formulated using CREKA and LyP-1 [108]. Additionally, PTX prodrugs were obtained using transferrin (Tf) and Fmoc-L-glutamic acid 5-tert-butyl ester (linker) to specifically target tumor tissues and cells [109]. Similarly, peptide-based prodrugs of PTX were also formulated such as the Tat-based self-assembling peptide, which is used to deliver PTX intracellularly [110], the tumor-homing cell-penetrating peptide (CPP) [111], and recombinant chimeric polypeptides (CPs) [112]. Additionally, PTX prodrugs can be obtained using small molecules such as docosahexaenoic acid (DHA) [113], conjugated linoleic acids (CLAs) [114], and oligo(lactic acid)_8_ [115]. Finally, hybrid prodrugs for PTX also exist, which are a combination of two drugs or more that is capable of producing synergistic effects, reducing the adverse effects related to a high dose of a single drug, and overcoming the multidrug resistance mechanism of cancer cells during treatment [116]. PTX hybrid prodrugs were delivered using other anticancer drugs such as doxorubicin (DOX) [117], camptothecin (CPT) [118], and the nucleic acid oligonucleotide [119].

### 4.5. Emulsions

Generally, macroemulsions are defined as the dispersion of one liquid in another liquid and it is considered a two-phase system [120]. They are turbid or opaque, viscus, and thermodynamically unstable, and their preparation is complicated as sheer is needed. On the other hand, microemulsions are translucent, thermodynamically stable, have a lower viscosity, and form spontaneously [121]. Based on the name, nano-emulsions should have a droplet size lower than microemulsions. As a matter of fact, nano-emulsions have a droplet size of 20–200 nm and a narrow particle size distribution [122,123,124].

A Tocosol^TM^ nano-emulsion was established early in 2000. It was formulated using an a-tocopherol isomer of vitamin E as a solubilizing agent for PTX and vitamin E TPGS as an emulsifier. Unfortunately, studies in phase III showed that the overall response rate was only 37%, while it was 45% with Taxol^®^. Based on that, the Tocosol^TM^ nano-emulsion was terminated [125]. Recently, Shakhwar et al. tried to reform a Tocosol^TM^ nano-emulsion using the c-tocotrienol (c-T3) isomer instead of a-tocopherol and the PEGylated c-T3 surfactant instead of vitamin E TPGS. Their results showed that the reformulated PTX was more active toward pancreatic tumor cell lines than the previous formulation [126].

Moreover, self-emulsifying drug delivery systems (SEDDSs) and self-microemulsifying drug delivery systems (SMEDDSs) are combinations of the non-aqueous components of emulsions and microemulsions, respectively [127], such as oils, surfactants, and if present, cosurfactant or cosolvents. These mixtures can be readily dispersed when diluted with an aqueous phase (gastric fluids) in the body and then spontaneously emulsified to form fine oil-in-water (O/W) microemulsions. This process can be sped up by slight mechanical agitation, and in vivo, this can be obtained by gastrointestinal motility [18,122,128]. A novel SMEDDS was developed for oral delivery of PTX, and it was administered to patients with advanced cancer and compared with orally administered Taxol^®^. The SMEDDS was co-administered with cyclosporin A to inhibit P-gp and CYP3A4. This formula was safe and well-tolerated by patients and had comparable bioavailability to oral Taxol^®^. In addition, the T-max of the SMEDDS was lower than the orally delivered Taxol^®^. This means that the absorption was higher in the novel formula, and this may be related to the added excipients [129]. In another study, the oral delivery of PTX was designed as an SEDDS. In this study, tocopheryl polyethylene glycol succinate was used to assist the emulsification. The results indicated that this system had higher G2M cell cycle arrest, apoptosis, mitochondrial membrane potential disruption, and ROS production in comparison with Taxol^®^. Moreover, the oral bioavailability of the SEDDS was about fourfold greater than Taxol^®^. Considerable reductions in the volumes and weights of the tumors were detected in syngeneic mammary tumors in SD rats. Additionally, this system was safe, stable, and caused low lung metastasis [130].

### 4.6. Implants

Drug-loaded polymeric implants are considered a pioneering approach in drug delivery. Active ingredients can be delivered to malignant cells using biodegradable polymers in continuous, sustained, and predictable patterns. Owing to their nature, biodegradable polymers do not need to be removed surgically after their application and thus eliminate complications associated with the long-term safety of implanted devices with non-biodegradable polymers. Additionally, the postsurgical local insertion of a biodegradable implant device loaded with an anticancer drug can avoid the further spread of cancer cells while avoiding toxic chemotherapy adverse effects in the patient [131]. Recently, an in situ depot-forming implant (ISFI) has been developed which can be injected as a liquid and then subsequently solidified [132,133]. In this way, an effective dosage form can be delivered with the avoidance of surgical insertion [134]. Moreover, ISFIs have relatively simpler preparation conditions and fewer complications than solid implants [135,136]. The PTX ISFI was formulated using PLGA to improve its efficiency and toxicity. This formula provided an in vitro sustained release of PTX for 28 days [137].

### 4.7. Nanocrystals

Nanocrystal formulations have become more attractive for the delivery of chemotherapies due to their superior properties in comparison with other nano-delivery approaches [138,139,140]. Nanocrystals eliminate the need for chemical carriers, therefore eradicating any toxic side effects induced by the excipients used for solubilization or coating and also providing about 100% drug loading, which ensures suitable concentrations of the drug even at low doses [141]. Additionally, due to the stable and uniform physical properties of crystalline particles, the enhancement of the pharmacokinetics and biodistribution properties of the anticancer drugs are anticipated [142,143,144,145].

Nanocrystals can be produced either by top-down or bottom-up methods. The top-down technique involves utilizing a high mechanical energy force to produce nanocrystals from large crystals by media milling or high-pressure homogenization [142,143]. These techniques are generally used to formulate insoluble drugs, especially those used for oral drug delivery [146,147]. In the high-pressure homogenization method, large drug crystals are forced across fluidic pressure and an impact valve, which leads the drug crystals to break down into tinier particles. The control of the particle size is achieved through the pressure and space among the impact valves. On the other hand, in media milling, the grinding of large crystals of the drug is obtained using solid particles like yttrium-stabilized zirconia, cerium, highly crosslinked polystyrene resin-coated beads, and stainless steel [142].

In the bottom-up approach, which involves the antisolvent perception method, nanocrystals can be produced directly from the drug solution. When the drug solution is mixed with an antisolvent with poor drug solubility, in such a case, the decrease in solubility leads to nucleation and crystal growth of the drug, and these are the two critical steps of this method [148]. As more nuclei form during the nucleation stage, then the growth of each nucleus is lower, and based on that, the nucleation step needs to be monitored carefully. The ultrasonic waves produced by sonication can help reduce the size of the nanocrystals by decreasing the particle agglomeration, achieved by breaking down the contact between particles. Consequently, perception and ultrasonication (PU) are commonly used in the bottom-up method [149,150].

The anti-solvent method produced nanocrystals with a smaller size that were cost-effective, simpler, and easy to scale up in comparison with other methods of the top-down approach [151,152]. However, various factors during nanocrystal preparation can be controlled to influence the size and morphology of nanocrystals obtained by the antisolvent method, such as the drug concentration, drug solution flow rate, temperature, solvent-to-antisolvent volume ratio, stirring speed, and the ultrasound wave characteristics [152,153,154]. In addition, the addition of surfactants and polymers during the crystallization process has an impact on the size or the shape of the drug’s nanocrystals [155]. This shows that engineering the modifications of nanocrystals according to our preference and usefulness is possible. Moreover, a combination of both approaches—the top-down and bottom-up methods—can also possibly obtain NCs with a smaller size (<100 nm), narrow distribution, and less production time [156]. In addition, the shape of nanocrystals is also considered important in controlling the activity and toxicity of anticancer drugs. For instance, the rod shape of some drug nanocrystals has superior anticancer activity and toxicity in comparison with the spherical shape [157]. Another study showed that the needle shape of some drug nanocrystals provides better accumulation in some cancers, which may be referred to as an increasing ability of these nanocrystals to be entrapped [144]. Moreover, the size of the nanocrystals is very critical for the in vivo performance of drugs. For instance, smaller nanocrystals have more dissolution rates than larger ones. Conversely, larger nanocrystals may provide sustained release behavior, which results in greater drug accumulation in tumors similar to drug depots. On the other hand, the smaller one is more stable because of the lower accumulation. Finally, the surface treatment or coating of nanocrystals with a polymer or surfactant can further improve the anticancer properties of the nanocrystals and the stability [10,158].

Manipulation during the preparation of the nanocrystals is possible and might end up in unexpected favorable outcomes. Therefore, this indicates the significance of controlling nanocrystals’ properties based on the efficiency, effectiveness, and safety of the anticancer drug, as these can be improved and manipulated indirectly during nanocrystal preparation, especially in the case of the nanocoating.

## 5. PTX Nanocrystals

As PTX is a hydrophobic drug with inferior water solubility properties, it is improved greatly by nanocrystal formulation [159]. Based on that, many studies employed the nano-crystallization techniques not only to improve the oral delivery of PTX, but IV, intraperitoneal (IP), and local and intertumoral delivery systems were also developed. Additionally, superior and interesting properties of the PTX NCs were achieved by performing additional modifications to the NCs, such as stabilizing them with surfactants and polymers or coating them with polymers. Table 1, Table 2 and Table 3 summarize these modified delivery systems by classifying them into three main categories, according to their route of administration: either IV (Table 1), oral (Table 2), or local and intraperitoneal (Table 3) delivery systems. Additionally, the summary tables shed light on the methods used in the preparation of these modified NCs, the in vitro or in vivo models used, and the advantages obtained based on the developed formulations. Nearly the majority of these NCs had a rode-like shape with drug-loading capabilities (>50%), and their size was between <50 and 500 nm (mainly 100–300 nm). The most common route of administration for these novel formulas was the intravenous (IV) route (Table 1), and the most common method of preparation was the antisolvent or precipitation method. The most common cancer cell lines or types of cancer tested were breast cancer (MCF-7 cell lines), followed by ovarian and then lung cancer. Finally, the aims for modifications were mainly focused on providing more solubility and tumor and cancer cell targeting, less elimination and side effects, and more anti-cancer effects with a smaller dose. In addition, it appears that the NCs’ formulation provided a suitable method for multiple drug combinations.

## 6. Future Aspects

It is worth mentioning that the nanocrystals are formed by weak, non-covalent interactions. This leads drug nanocrystals to continue to dissolve, albeit slowly, when in contact with water. As such, any surface-coated materials on drug nanocrystals will eventually be detached during the dissolution process. This not only makes it a challenging task to develop surface-treated nanocrystals but also results in transient target-homing effects.

In this regard, the concept of hybrid nanocrystals may overcome this limitation by physically integrating guest molecules among the crystal lattices of nanocrystals. Small molecules such as fluorescent dyes have been demonstrated in vitro and in vivo of paclitaxel nanocrystals. It is thus possible to utilize larger molecules as a guest in making hybrid nanocrystals.

Finally, it is pertinent to understand and eventually predict drug release and dissolution kinetics of paclitaxel nanocrystals in a biological environment. This may be aided by in vitro experimentation and physics-based simulation. One ultimate goal in developing paclitaxel nanocrystals is precision medicine for cancer treatment, which can only be enabled by a thorough understanding of the interactions and the pharmacokinetic characteristics of drug nanocrystals in tissues and cells.

## 7. Conclusions and Remarks

Several delivery systems for paclitaxel drugs have been developed to enhance their solubility and pharmacological properties. Of these delivery systems, nanocrystal formulations are considered a promising modality that can also have the advantage of providing a suitable platform for surface modifications. Based on that, many studies employed nano-crystallization techniques not only to improve the oral delivery of PTX but also to improve the IV, intraperitoneal (IP), and local and intertumoral delivery systems, where the applications of surface modifications can be of greater value in terms of targeted delivery. Moreover, these systems can provide 100% loading and releasing capacities for the drugs as well as gain the advantages of being formulated as particles that have different circulation patterns, fates, cellular uptake mechanisms, and sometimes preferable efficacy and safety profiles compared with free drugs. Finally, more studies are needed to understand the molecular basis for the formation and interaction of these nanocrystals with biological systems, and consequently providing better platforms for useful modifications in the future.

## Figures and Tables

**Table 1:** Modified PTX NC formulations for intravenous (IV) drug delivery.

PTX NC	Method of Preparation	The Models Used and the Reference or Control Formula	Benefits, Aims, and Other Notes	Refs.
Albumin-coated PTX-NC (Alb-PTX NCs)	NC crystallized in the medium containing Pluronic F-127 and then coated with albumin “Cim-F-alb”	The new formula was compared to Abraxane and solvent-dissolved PTX In vitro models including Biolayer interferometry analysisCell culture models: J774A.1 macrophages and SPARC^+^ B16F10 melanoma cells In vivo model: mouse model of B16F10 melanoma	High drug loading (90%) and serum stability Equivalent cytotoxicity. More stability in undiluted serum. Less interaction with serum proteins. In cell culture studies, demonstrated suitable cell interaction profiles (depressed uptake by macrophages and great uptake by melanoma cells). In the in vivo studies, exhibited prolonged plasma t_1/2_ and superior accumulation in tumors by about 1.5 and 4.6 times, respectively. Exhibited superior antitumor efficacy.	[160,161]
Surface modified PTX-NCs with apo-transferrin (Tf) or hyaluronic acid (HA)	PTX NCs were prepared by the nanoprecipitation Method, and then the surface was modified by grafting with Tf or HA	The new formula was compared to PTX-NC and pure PTX drug In vitro models: drug release in PBS with or without tween 80 Cell culture models: HaCaT normal cells and MCF-7 cancer cells	PTX release was faster. Improve the cellular uptake, permeability, and cell growth inhibition (60%) against the cancer cells. The effect on the normal cells was inferior. Provide targeted delivery to cancer cells.	[162]
Hyaluronic acid (HA) coated PTX NCs	The NCs were prepared by the top-down method using homogenization	The new formula was compared to Taxol^®^ and heparin-coated PTX NCs In vitro models: 2D monolayer and 3D spheroids Cell culture models: MDA-MB 231 cells In vivo model: LA-7 tumor-bearing rat model	Exhibited superior in vitro efficacy. HA-PTX NCs incur receptor-mediated endocytosis by binding to CD44 receptors. The in vivo studies indicated significantly prolonged blood circulation time of PTX. Exhibited superior efficacy with reduced lung metastasis and toxicity.	[163]
PEGylated PTX NCs	The NCs were prepared by the antisolvent precipitation method combined with probe sonication	The new formula was compared to PTX NCs and Taxol^®^ In vivo model: breast cancer xenografted mice model and a model of lung tumor metastasis quantified by the luciferase activity	Superior stability under both storage and physiological conditions. In vivo studies showed significant improvement of the antitumor activity in facing in situ or metastatic tumors.	[164]
PEGylated polyelectrolyte multilayer-coated PTX NCs	The layer-by-layer method was used to coat PTX NCs with alternating layers of oppositely charged polyelectrolytes, utilizing a PEGylated copolymer as the upper layer, and PTX NCs were prepared by a wet milling approach	The new formula was compared to Abraxane and PTX NCs In vitro models: physiologically relevant media and human RBC hemolysis Cell culture models: HT-29 cells In vivo model: NMRI-nu mice bearing HT-29 subcutaneous xenografts	Slowed down the dissolution. Offered colloidal stability in physiologically simulated media. Showed no innate effect on cell viability using HT-29 cells. No hemolytic activity was detected. Quickly eliminated from the bloodstream and accumulated in the liver and spleen (mononuclear phagocyte organs). Poor tumor accumulation.	[165]
PTX NCs modified with PEG and folic acid (FA)(PTX NCs-PEG-FA)	PTX NCs were prepared by thin-film hydration method, which is a bottom-up method, and then modified with both PEG and FA derivatives using thin-film hydration technique	The new formula was compared to Taxol^®^, PTX NCs, and PTX NCs-PEG In vitro models: plasma Cell culture models: 4T1 breast cancer cells In vivo model: PK rat model and 4T1 orthotopic breast cancer-bearing nude mice	More size stability in plasma. Improved cellular uptake and growth inhibition in cells. An in vivo pharmacokinetic study showed a significant increase in the circulation of PTX. In vivo cancer model showed that it significantly enhanced the accumulation of PTX in the tumor and effectively inhibited tumor growth.	[166]
Surface hybridization of PTX NCs by DSPE-PEG 2000	PTX NCs were prepared by anti-solvent method, and DSPE-PEG 2000 was incorporated by hybridization	The new formula was compared to PTX solution and PTX NCs In vitro models: in vitro release study In vivo model: PK rats’ model	Similar size with an increased negative charge. The in vitro study showed that the release of PTX was significantly slower. The pharmacokinetics studies showed a greater area under the curve (AUC) and a lower clearance rate.	[167]
Cube-shaped PTX NC prodrug with surface functionalization of SPC and MPEG-DSPE	PTX was labeled with fluorophore conjugate 4-chloro-7-nitro-1, 2, 3-benzoxadiazole (NBD-Cl) (PTX-NBD), which was synthesized by a nucleophilic substitution reaction of PTX with NBD-Cl in high yield. The PTX-NBD NCs were prepared by the anti-solvent method followed by surface functionalization of SPC and MPEG-DSPE.	The new formula was compared to free PTX-NBD and the sphere-shaped PTX-NBD nanocrystals with surface functionalization of SPC and MPEG-DSPE (PTX-NBD@PC-PEG NSs) Cell culture models: HeLa cells	The cube-shaped PTX-NBD@PC-PEG NCs exhibited better drug loading and stability properties. It showed a remarkable decrease in burst release, efficiently enhanced cellular uptake, and had a better ability to kill cancer cells in vitro using HeLa cells. These NCs can be useful for cell imaging and chemotherapy.	[168]
Surface-modified PTX with positively charged poly(allylamine hydrochloride) (PAH)	Nano-precipitation method (bottom-up approach) was employed to prepare PTX NCs, and the surface-modified NCs were obtained by an absorption method with the positively charged polymer	The new formula was compared to pure PTX, PTX NCs, and negatively charged poly (sodium 4-styrene sulfonate) PSS PTX NCs In vitro models: PBS (pH 7.4) containing 0.5% (w/v) tween 80 and bovine serum albumin (BSA) Cell culture models: A549 cells	Higher drug release. Stronger interaction with bovine serum albumin. Greater cellular internalization, uptake, and cytotoxicity.	[169]
A non-covalent transferrin-stabilized PTX NCs	The NCs were prepared by the antisolvent precipitation method augmented by sonication	The new formula was compared to PTX solution, PTX NCs, and Taxol^®^ Cell culture models: human KB epidermal carcinoma cells and SKOV-3 ovarian cancer cells In vivo model: mice inoculated with KB cells	The in vivo efficacy studies on KB-bearing mice showed a significantly superior tumor inhibition rate compared with PTX NCs and less efficacy than Taxol, but with a better toxicity profile. However, in cellular models, it showed similar efficacy 72 h after treatment.	[158]
PTX NCs stabilized by D-α-tocopheryl polyethylene glycol 1000 succinate (TPGS)	The NCs were prepared by three-phase nanoparticle engineering technology (3PNET)	The new formula was compared to Taxol^®^ and PTX/Pluronic F127 (F127) NCs Cell culture models: P-glycoprotein-overexpressing PTX-resistant (H460/TaxR) cancer cells In vivo model: PK using CD-1 mice	The greater the amount of TPGS in the formula, the greater cytotoxicity and cellular internalization. TPGS PTX NCs demonstrated a significantly sustained and prolonged in vitro release pattern. PK studies indicated more rapid clearance. However, they were more effective in promoting the accumulation of PTX in drug-resistant tumors.	[170]
Herceptin (HCT)-functionalized PTX NCs	PTX NCs were prepared by sono-precipitation approach, and then HCT was coated, applying a facile non-covalent technique	The new formula was compared to PTX NCs and PTX powder In vitro models: release study Cell culture models: HER2-positive breast cancer cell lines	Exhibited a sustained release pattern comparable to PTX NCs. Demonstrated a higher binding affinity, greater cell-specific internalization, and inhibition of growth to HER2-positive breast cancer cell lines.	[171]
PTX-NCs coated with Pluronic^®^ F68 (PEG-PPG-PEG block polymer)	The NCs were prepared by the anti-solvent method	The new formula was compared to Taxol^®^ and PTX NCs In vivo model: tumor-bearing (HT-29 and KB cells) mice and female nude outbred mice	These NCs exhibited similar or better antitumor efficacy and lower toxicity in comparison with Taxol. The in vivo study showed a significant enhancement in the blood circulation of PTX and accumulation in tumor tissue. However, the definite amount that reached the tumor was still minimal for the administered dose. The maximum amount of the coated NCs was significantly obtained in the liver compared with the other organs relative to the uncoated PTX NCs.	[172]
Triphenylphosphonium (TPP^+^)-stabilized PTX NCs (TPP^+^ PTX NCs)	Precipitation-resuspending method	The new formula was compared to free PTX and unmodified PTX NCs In vitro cell culture models: 2D monolayer and 3D multicellular spheroids (MCs) of MCF-7 cells and MCF-7/ADR cells	A mitochondria-targeted system was developed. Showed the strongest cytotoxicity that was associated with a reduction in mitochondrial membrane potential. Showed greater penetration and superior growth inhibition.	[173]
Platelet membrane-coated or cloaked PEG-PTX NCs (PPNCs)	The modified emulsion-lyophilized crystallization method	The new formula was compared to PTX NCs Platelet aggregation was examined using a spectrophotometric method In vitro drug releasee Cell culture models: 4T1 breast cancer cells In vivo model: BALB/c mice injected with 4T1 cells model	Minor risk of thrombus formation after injection was observed. Higher cellular uptake and greater cytotoxicity. In vivo studies showed the ability to deliver a higher dose of the drug and target the site of the coagulation (surgery or vascular disrupting), which improved the antitumor efficacy and decreased toxicities.	[174]
RGD peptide -PEGylated PTX NCs coated by polydopamine (PDA) (NC@PDA-PEG-RGD)	The NCs were prepared using modified antisolvent–sonication method	The new formula was compared to free PTX, PTX NCs, PTX NCs-PEG, and PTX NCs-PDA-PEG In vitro models: plasma for size stabilityCell culture models: A549 lung cancer cell line In vivo model: nude mice A549 bearing cancer model	More size stability in plasma. Showed superior cellular uptake, growth inhibition, and cytotoxicity on A549 lung cancer cell line. In vivo demonstrated significantly greater accumulation in the tumor and slower tumor growth.	[175]
PTX and lapatinib (LAPA) composite nanocrystals with PDA and PEG modification(cNC@PDA-PEG)	PEG coat was introduced into the cNC via PDA) coat to get PEGylated composite NCs (cNC@PDA-PEG). The NCs were prepared using the bottom-up method or precipitation-resuspending method.	The new formula was compared to free PTX and unmodified PTX NCs In vitro models: plasma and blood Cell culture models: MCF-7/ADR cancer cells	cNC@PDA-PEG had optimum size and stability. The in vitro release study showed that both PTX and LAPA were released completely from cNC@PDA-PEG in 3 days, while only 30% of the drug was released from bulk drugs or unmodified NCs. Showed negligible hemocytolysis and improved therapeutic effect on MCF-7/ADR through endocytosis of whole NCs.	[176]

**Table 2:** Modified PTX NC formulations for oral drug delivery.

PTX NC	Method of Preparation	The Models Used and the Reference or Control Formula	Benefits, Aims, and Other Notes	Ref.
Pluronic-grafted chitosan as a stabilizer for PTX NC (Pl-g-CH PTX NCs)	A novel Pluronic-grafted chitosan copolymer was established and then utilized as a functional stabilizer for PTX NCs. Generally, the NCs were prepared using a high-pressure homogenizer.	The new formula was compared to Taxol^®^ Cell culture models: Caco-2 cells and B16 F10 murine melanoma cells In vivo model for oral PK evaluation: Wistar ratsIn vivo model for efficacy study: healthy Balb/C mice injected with B16 F10 murine melanoma model	Improving intra-cellular accumulation. Improving the absorption by the transcellular and paracellular routes. Showed a P-gp inhibitory property. The in vivo model demonstrated more anti-tumor efficacy and growth reduction after oral delivery, and this was related to the enhancement in the systemic circulation as both the absorption and bioavailability were improved significantly.	[177]
PTX NCs stabilized by tween 80 or low molecular weight synthetic polymer sodium polystyrene sulfonate (PSS)	The top-down method was performed using a microfluidizer as a high-pressure homogenizer that was used to prepare the NCs without using any organic solvent	The new formula was compared to formulas stabilized with high molecular weight polymers glycol chitosan (GC) and sodium alginate (SA), as well as with PTX solution and PTX-NCs Cell culture models: MCF7 and MDA-MB breast cancer cell lines In vivo model: PK in male Wistar rat model	The prepared NCs were more suitable, efficient, and exhibited a considerable increase in the dissolution rate, which indicated an enhancement in its bioavailability. The in vitro cell culture study showed more efficiency and potency in killing and inhibiting the growth of the cancer cells. In vivo pharmacokinetic studies demonstrated a considerable increase in AUC_0–t_, C_max_, and MRT and a decrease in T_max_.	[178]
Transferrin (TF)-modified PTX NCs	PTX NCs were prepared using the precipitation–resuspension method	The new formula was compared to Taxol^®^ and unmodified PTX NCs In vitro models: in situ intestinal perfusion study Cell culture models: Caco-2 cells and MCF-7 cancer cells In vivo model: PK Sprague Dawley rat model	Showed an enhancement of cellular monolayer penetration. Had superior suppression in MCF-7 cell growth. Showed an enhancement of intestinal absorption. The pharmacokinetic studies also demonstrated greater C_max_ and AUC than both PTX NCs and Taxol^®^ while having the lowest t_max_.	[179]
Poly(sodium pstyrenesulfonate) (PSS)-modified PTX NCs	Not mentioned	In vitro models: interactions with biomolecules in oral delivery pathways Cell culture models: Caco-2 cell lines	Suitable mono-dispersion and stability in the gastrointestinal tract (GIT) environments for at least 24 h. No substantial interactions with pepsin or trypsin enzymes were detected in the GIT environments. PSS-modified PTX NCs passed through the mimical intestinal epithelial cell (Caco-2 cell lines) with about 25% transmittance. However, the concentration of the NCs should be controlled to avoid toxic effects on the cells.	[180]

**Table 3:** Modified PTX NC formulations for local and intraperitoneal drug delivery.

PTX NC	Route of Administration	Method of Preparation	The Models Used and the Reference or Control Formula	Benefits, Aims, and Other Notes	Ref.
PTX NC-loaded PECT hydrogels	Local delivery and peritumoral administration	PTX NCs were prepared by three-phase nanoparticle engineering technology (3PNET), while PTX-NC-based PECT (PTX-NC-PECT) gel was prepared based on the “cold” method	The new formula was compared to a nanoparticle-based system (PTX-NP-PECT) and controlled hydrogel of Pluronic^®^ F127 In vitro models: release study In vivo model: MCF-7 tumor-bearing mouse models	High loading capacity of the drug. In vitro release was more effective and homogeneous. In vivo near-infrared fluorescence (NIRF) imaging indicated the ability to maintain the payloads of 1,1-dioctadecyltetramethyl indotricarbocyanine iodide (DiR) at a peri-tumoral site for about 21 days. Exhibited the most complete release system with the greatest anti-tumor efficacy and apoptosis effect.	[181]
Silica-coated PTX NCs Si	Intra-peritoneal (IP)	Precipitation–resuspending method	The new formula was compared to uncoated PTX-NC or Abraxane Cell culture model: neural stem cells and OVCAR-8 cells In vivo model: athymic nude mice which inoculated with 2 M OVCAR-8.eGFP.ffluc human ovarian cancer cells	More effective in loading neural stem cells (NSCs). In vivo studies showed that loaded NSCs preserved their migratory ability and, for low PTX dose, were more effective against ovarian tumors.	[182]
Poly-tannic acid-coated PTX NCs (PTA-PTX NCs)	Intertumoral injection	The NCs were prepared using the thin-film hydration method followed by probe sonication	The new formula was compared with or without laser irradiation to PTX Cell culture models: 4T1, A549, and HepG2 cells In vivo model: 4T1 tumor-bearing mice	PTX NCs were prepared to act as a chemo-therapeutic agent and poly-tannic acid (pTA)-coated PTX NCs in the presence of Fe3+ acting as a potential agent for photothermal therapy (PTT). The cellular uptake was significantly improved. A synergistic effect with laser irradiation was observed. Demonstrated mild photothermal effect in vivo and the greatest effect in tumor inhibition upon laser irradiation.	[183]
PTX NC with F127 hydrogel	Intertumoral injection	Precipitation–resuspending method. The cold method was used for hydrogel preparation.	The new formula was compared to PTX or PTX microcrystal-based hydrogels In vitro erosion of the hydrogels and drug release In vivo model: 4T1 tumor-bearing BALB/c mice	PTX NCs gel offered optimum properties with high drug loading combined with moderate drug release and erosion profiles. Superior anti-tumor efficacy in 4T1 tumor-bearing BALB/c mice.	[184]
In situ cross-linkable hydrogel depot containing PTX NCs	Intraperitoneal (IP)	Anti-solvent and temperature-induced crystallization method	The new formula was compared to Taxol^®^ and microparticulate PTX precipitates (PPT) Cell culture models: SKOV3 cells In vivo model: healthy Balb/c mice for toxicity studies and Balb/c mice (SKOV3-Luc) cell-bearing mice for the efficacy study	Superior killing efficiency and more toxicity in SKOV3 cell line. The in vivo study indicated improved dissolution, cellular uptake, and lower maximum tolerated dose. It also showed that a single IP dose was sufficient in extending the survival of tumor-bearing mice.	[185]
PTX-NCs combined with niclosamide (NLM) NLM-NCs co-loaded PLGA-PEG-PLGA thermosensitive hydrogel (PN-NCs-Ts)	Intratumoral injection	PTX-NCs were prepared by the “3PNET” method	The new formula was compared to PTX-NCs, PTX-NCs-Ts Gel, NLM-NCs, NLM-NCs-Ts gel, and PN–NCs-Ts gel In vitro drug release Cell culture models: MDA-MB-231 cells In vivo model: BALB/c nude mice inoculated with MDA-MB-231 cells	Sustained and significantly delayed drug release both in vitro and in vivo. The combination with NLM improved PTX cellular uptake, apoptosis, and provided inhibition of cell migration. The in vivo studies showed significant inhibition of tumor growth with acceptable safety and effectively overcoming it. Triple-negative breast cancer (TNBC) progress and drastically prevented breast cancer stem cells (BCSCs).	[186]

## Data Availability

Not applicable.
